# Astrocytes as Context for the Involvement of Myelin and Nodes of Ranvier in the Pathophysiology of Depression and Stress-Related Disorders

**DOI:** 10.20900/jpbs.20230001

**Published:** 2023-02-22

**Authors:** José Javier Miguel-Hidalgo

**Affiliations:** Department of Psychiatry and Human Behavior, University of Mississippi Medical Center, 2500 North State Street, Jackson, MS 39216, USA;

**Keywords:** astrocytes, oligodendrocytes, depression, stress, myelin, connectivity, white matter

## Abstract

Astrocytes, despite some shared features as glial cells supporting neuronal function in gray and white matter, participate and adapt their morphology and neurochemistry in a plethora of distinct regulatory tasks in specific neural environments. In the white matter, a large proportion of the processes branching from the astrocytes’ cell bodies establish contacts with oligodendrocytes and the myelin they form, while the tips of many astrocyte branches closely associate with nodes of Ranvier. Stability of myelin has been shown to greatly depend on astrocyte-to-oligodendrocyte communication, while the integrity of action potentials that regenerate at nodes of Ranvier has been shown to depend on extracellular matrix components heavily contributed by astrocytes. Several lines of evidence are starting to show that in human subjects with affective disorders and in animal models of chronic stress there are significant changes in myelin components, white matter astrocytes and nodes of Ranvier that have direct relevance to connectivity alterations in those disorders. Some of these changes involve the expression of connexins supporting astrocyte-to-oligodendrocyte gap junctions, extracellular matrix components produced by astrocytes around nodes of Ranvier, specific types of astrocyte glutamate transporters, and neurotrophic factors secreted by astrocytes that are involved in the development and plasticity of myelin. Future studies should further examine the mechanisms responsible for those changes in white matter astrocytes, their putative contribution to pathological connectivity in affective disorders, and the possibility of leveraging that knowledge to design new therapies for psychiatric disorders.

## INTRODUCTION

There is a solid body of experimental and human postmortem brain evidence demonstrating that astrocytes play fundamental roles in supporting neuronal metabolism, survival and communication at synapses, and in the development and maintenance of the white matter. These essential roles and the ability of astrocytes to become reactive when confronted with injury or disease are reflected in their crucial involvement in the neuropathological mechanisms of most neurological and neurodegenerative disorders [1–3]. The recent decades have also witnessed increasing research attention towards the role of white matter pathology in brain connectivity changes associated with depression and other affective psychiatric disorders [4–7]. Connectivity involves the exchange of information between brain regions that is reflected in correlated functional changes [8,9] and also in structural alterations in the bundles of axons that actually propagate the signals to connect distant brain regions [10–12]. Part of the connectivity alterations in psychopathology can be accounted by local changes in synaptic transmission and cell metabolism in the gray matter (GM) [13–15]. These alterations will eventually result in undesired or maladaptive signal patterns that could simply be conveyed by axons in the WM to other brain destinations, so spreading dysfunction. In addition, regardless of neuronal, synaptic and glial [16] disturbances in the GM, the propagation of signals within the WM could be altered by pathology of the intrinsic WM components affecting the myelin insulation of axons or the structure and molecular composition of the axons themselves [17–20]. In fact, alterations intrinsic to the WM are now considered to be part of plastic processes occurring during learning and other normal non-pathological plastic processes in the brain [21]. Even in non-diseased brain structures, many studies have shown that the myelin that wraps many axons in the WM can undergo substantial morphological and neurochemical plastic changes that contribute to connectivity modifications leading to actual behavioral and cognitive adaptations, including significant changes in learning and memory [22,23]. Human postmortem studies in depression-diagnosed subjects and in animal models of potent risk factors for depression, such as stress, have also identified striking effects on the abundance and morphology of oligodendrocytes and myelin or their markers in some relevant brain regions [17,18,24–29]. Both in the gray and white matter several lines of research have shown that astrocytes appear to be seminal to basic cellular processes in neurons and myelin-forming oligodendrocytes that support connectivity [16,18,20,30,31]. In the following, there is a review of some major aspects of astrocyte neurochemistry and function that support those processes, with particular emphasis on astrocyte involvement in ensuring adequate conduction of nervous signals in the white matter.

## GM ASTROCYTES INVOLVED IN PROCESSES AFFECTING CONNECTIVITY IN DEPRESSION AND STRESS

**Figure 1.**
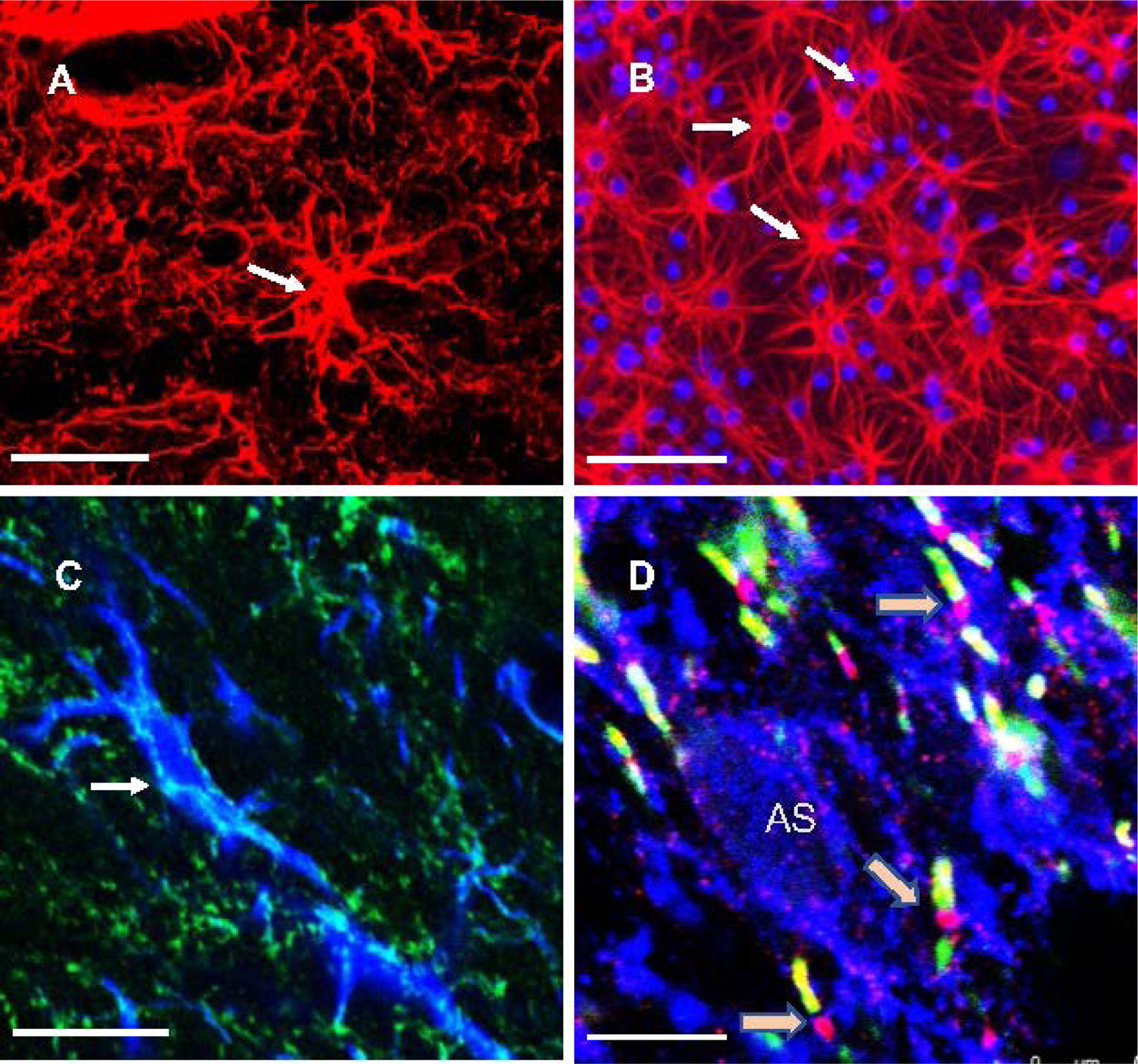
Immunolabeled astrocytes in histological sections from the prefrontal cortical regions of rats (A, C, D) and from mixed-cell (astrocyte-neurons-oligodendrocytes) primary cultures from rat frontal cortex (B). Arrows in A, B and C point to the cell bodies of astrocytes. Red immunofluorescent label in A and B corresponds to astrocyte cytoskeletal marker GFAP. Cell nuclei in B are stained blue with nuclear marker DAPI. (C) GFAP labeling of astrocytes (blue) coexists with green label for glutamate transporter EAAT1 in cell bodies (arrow) and processes of astrocytes. D) White matter astrocytes display significant amounts of EAAT1, which was immunolabeled here to display blue fluorescence together with markers that identify nodes of Ranvier (beige arrows). Nodes of Ranvier are identified by using antibodies to neurofascin (red/pink) and to paranodal protein CASPR (green/yellow). Note that NRs are in close proximity to EAAT1-positive processes (blue immunofluorescence). “AS” denotes the nucleus of an EAAT1-positive astrocyte cell body in the white matter. Calibration bars at bottom left of each micrograph: (A) 50 μm, (B) 100 μm, (C) 15 μm, (D) 5 μm.

Each astrocyte and its highly branched processes occupy a region of the gray matter that is adjacent to neighboring astrocytes territories with very little overlap [32] (Figure 1), each astrocyte extending up to 200 μm in diameter in humans [33]. The linear reach of the processes is thus small relative to the reach of axons, although the influence of astrocyte cytoplasmic changes is somewhat extended by the gap junctions between astrocytes that permit cytoplasm to cytoplasm communications through shared transient intracellular calcium increases and the exchange of molecules no larger than 1.5 kdalton. Nevertheless, the reach and speed of these astrocyte-to-astrocyte communication channels are respectively limited and slow, so that long range connectivity is rather based in the long processes of neurons and their ability to speedily send action potentials that spearhead synaptic communication between widely separated brain regions. The contribution of astrocytes to long range communication does not depend thus on their individual reach within and among networks, but rather on the ability of astrocyte processes to regulate connectivity at the sites of neurotransmitter release, or locally support the metabolism of neurons through their ability to generate processes that branch out and produce leaflets closely apposed to other neural elements such as synapses or capillaries [34]. Even when glial cell communication may affect glial and neuronal activity in small local circuits, the main influence of astrocytes on longer range connections most likely depends on the function of astrocytes in reuptake of neurotransmitters glutamate and GABA, although other aspects of astrocyte function such as providing metabolic and neurotrophic substrates to neurons may also have an indirect permissive role for connectivity by sustaining ATP production and allowing dendritic and spine plasticity that are critical to maintenance and recovery of membrane potential and to synaptic release adaptations in neurons. The tips of the highly branched processes of astrocytes in the GM enwrap or are closely associated with presynaptic and their corresponding postsynaptic neuronal elements [34]. The pervasive presence of these trios of cellular elements (presynaptic-postsynaptic-astrocytic) in the gray matter, tightly undergirding synaptic communication, has given place to the concept of tripartite synapse. This arrangement of astrocyte processes allows singular astrocytes to affect as many as 100,000 synapses through the tips of their highly-branched processes [35,36]. In addition to influencing connectivity at the synaptic level, tips of astrocyte processes in the gray matter also closely appose nodes of Ranvier and the adjacent paranodal regions (Figure 1D) along myelinated portions of afferent and efferent axons that respectively come from or enter the white matter, so that part of the influence of gray matter astrocytes on connectivity may depend on their role at nodes of Ranvier in segments of axons residing in GM. However, the majority of astrocytes subserving NRs are expectedly located in the white matter [37], where they would exert critical permissive and regulatory roles for the propagation of action potentials [38], which are the signals carrying most of the information allowing for efficient mid- and long-range connectivity. Thus, the involvement of WM astrocytes in processes influencing normal connectivity will be described and discussed in the next section.

## WM ASTROCYTES INVOLVED IN PROCESSES AFFECTING CONNECTIVITY IN DEPRESSION AND STRESS

WM astrocytes are morphologically different from the large population of protoplasmic astrocytes in the GM, and are termed fibrous astrocytes [33,39]. Processes stemming from WM astrocytes are in overall thicker, fewer and less branched than those in the GM, although recent studies have shown and recognized the possibility of distinct morphological variation in WM astrocytes, particularly in response to WM injury [40]. In addition, unlike in GM, processes of WM astrocytes tend to intermingle in the WM without forming mutually exclusive territories [33]. Some of their branches abut the basal lamina around blood vessels to support the blood brain barrier and allow for regulated exchanges of water and metabolites with the blood circulation. In addition, many of the astrocytes’ branches directly establish gap junctions with neighboring astrocytes and, importantly, with the cell membranes of oligodendrocytes. These gap junctions are formed by various types of connexins that are different in those between astrocytes as compared to those contacting astrocytes with oligodendrocytes. Various functions have been proposed for these cytoplasm-to-cytoplasm contacts, such as siphoning released extracellular potassium from axons to astrocytes, and eventually to the blood circulation, with the mediation of oligodendrocytes [41,42]. The critical role of gap junctions between astrocytes and oligodendrocytes to myelin maintenance is demonstrated in significant myelin disruptions observable in animal models deficient in one or more of the gap junction-forming connexins in astrocytes or oligodendrocytes [43–47]. Comparable alterations of astrocyte-oligodendrocyte gap junction-based communication may also contribute to the pathology of depression, since it has been found that gap junction coupling between astrocytes and oligodendrocytes may be reduced in the anterior cingulate cortex of subject with depression dying by suicide [48].

Altered structure and function of astrocytes and oligodendrocytes, and disturbed interactions between them have been proposed to play a major role in the pathophysiology of mood disorders, particularly MDD [49,50], while astrocyte atrophy is also being recently recognized to significantly occur during aging and neurodegeneration [51,52]. Low density of those cell types and reduced expression of astrocyte- and oligodendrocyte-specific gene transcripts were reported in postmortem human brains in MDD [17,19,20,53,54]. Similar features are observed in rodent models of depressive-like phenotypes caused by prolonged stress exposure such as chronic unpredictable stress [55–58]. However, most of these studies first focused on cortical gray matter and only later started to turn their attention to an involvement of white matter (WM) glial pathology as major contributor to functional and connectivity disturbances in MDD and models of stress-induced psychiatric disorders. Recently, prominent reductions in astrocyte density and astrocyte-specific protein and mRNA in ventral prefrontal WM in MDD [20,59] have been reported. In the same brain region, oligodendrocyte size decreases and low myelin-specific gene expression was detected in WM of human subjects [17,19]. These findings align with reports of reduced fractional anisotropy (measure of axon bundles’ integrity) in prefrontal WM [60] and corpus callosum [61] in MDD.

## ASTROCYTES AT NODES OF RANVIER

In addition to gap junction-mediated interactions with myelinated axons, the tips of a considerable proportion of WM astrocytic processes end in close apposition with nodes of Ranvier [38] (**NRs**), which are stretches of bare axon carrying ion channels for action potential regeneration and propagation. This closeness to NRs (Figure 1D) appears to be of great importance since more than 95% of NRs in WM are intimately associated with the ends of astrocytic processes (processes from another glial cell, NG2 cells, are also associated with NRs often in conjunction with astrocytes’ processes) [37]. Astrocyte processes and partially unpacked portions of the oligodendrocyte-generated myelin coat around the axon (dubbed paranodes) are the main glial structures around NRs [37,62]. To stabilize the structure of NRs, and allow for focused aggregation of voltage gated at nodes of Ranvier, there are specialized cell-adhesion components of the oligodendrocyte membrane. These components face other adhesion components in the axonal membrane at each of the paranodes delimiting both ends of the NR [62,63]. Astrocytes have been proposed to regulate the speed of action potential propagation by influencing the separation between the paranodes surrounding the NR [38,63,64]. In addition, there is a distinct set of axonal membrane proteins anchored at the node itself, including neurofascin and contactin, that form a cell adhesion apparatus directly interacting with voltage-gated sodium channels [65,66]. At NRs, neurofascin 186, one of the cell adhesion proteins, in turn serves also as docking point for various extracellular matrix components such as proteoglycans and Tenascin-R, the precise functions of which are starting to be elucidated. Astrocytes provide a major contribution to these extracellular components, although oligodendrocytes, NG2 cells and the axons themselves contribute to the release of some of the particular proteoglycans or cell adhesion factors [67]. In fact, some of the extracellular proteoglycans such as proteoglycan BRAL-1 are heavily produced by astrocytes, and significant quantities of phosphacan, versican, brevican, neurocan and tenascin R are contributed by astrocytes as well [67–70].

Interaction between astrocyte processes and axons at NRs is mediated by those extracellular matrix (ECM) components, which include the proteoglycans brevican, phosphacan, versican-2, and BRAL-1, as well as the glycoprotein tenascin-R, and the axonal adhesion molecules neurofascin-186 (NF186) and contactin [62,71]. Despite detected alterations in astrocyte processes in GM and WM in depression much remains to be known about how the ubiquitous astrocyte processes at NRs and the associated ECM molecules (ECMMs) and axon proteins are disturbed, and the role of those disturbances in connectivity changes. In a preliminary study in human PFC WM in MDD we found decreased levels of mRNAs for versican2, tenascin-R and NF186 and some of these alterations were still significant after removing outliers. Density of WM fibrous astrocytes has been found also decreased in CUS rats and in humans with MDD [20]. More recently we detected dramatic reductions in NR length and increases in the content of proteoglycan phosphacan associated with MDD and with chronic unpredictable stress in a rat model [72]. Thus, there appears to be significant pathology associated with WM astrocytes processes, astrocyte-linked ECMMs and axonal proteins at NRs. NR proteoglycans and tenascin-R bind to NR axon membrane proteins, forming a complex linking the axon cytoskeleton to the ECM. The link supports aggregation of axonal voltage-gated sodium channels (Na_v_s) at NRs during NR formation and maintenance [73,74]. As stated above, astrocytes variably express all of these molecules [67]. Astrocyte processes matter to WM function because they are a major brevican, phosphacan and BRAL-1 source and these molecules allow for versican-2 and tenascin-R anchoring, which in turn bind to axonal adhesion proteins (NF186 and contactin) that regulate Na_v_s’ clustering [73]. Accordingly, proteoglycan-tenascin-R-NF186 complexes are thought crucial to support action potential regeneration at NRs for subsequent propagation. Thus, reduced or disrupted involvement of astrocytes processes in the generation of ECM components or in their display to support extracellular adhesion interactions may lead to alterations of signal propagation along myelinated axons and underlie a mechanism contributing to disturbed PFC connectivity in depression and other affective disorders.

## REGULATION OF GLUTAMATE ACTIONS IN THE WHITE MATTER

Although neuron-to-neuron synapses supporting neurotransmitter-based communication are mostly absent in the white matter, substantial glutamate release in the white matter has been detected [75]. This release is proposed to regulate the activity of glial cells, namely oligodendrocyte precursor cells (OPCs, also known as NG2 cells), since the remarkable discovery was made that synapses between presynaptic axons and postsynaptic OPC occur in the white matter [75], and that increased activity actually regulates the differentiations of OPC into oligodendrocytes to produce new myelin in a form of activity-dependent myelin plasticity [23]. WM astrocytes would play and important role in this type of plasticity because they express high levels of excitatory amino acid transporter 1 (EAAT1; in rodents the homologous protein is also called GLAST) glutamate transporters [37,76,77] (Figure 1C) that would facilitate reuptake of released glutamate and thus contribute to terminate the actions of WM extracellular glutamate, indirectly regulating the connectivity by controlling the activity of OPCs. Postmortem human research in the brains of MDD patients has detected a reduction on the GFAP processes of astrocytes [20] and in size of CNPase-positive oligodendrocytes in the prefrontal white matter [78]. More recently low levels of GLAST proteins have been also measured in the ORB of human subjects with MDD [59] suggesting the possibility that a deficit of astrocyte processes or expression of EAAT1 may lead to a dysregulation of glutamate actions in the white matter.

Recent findings on the molecular composition of astrocyte processes suggest a role for ECMMs and EAAT1 transporters in regulating extracellular glutamate actions in the WM [71,79,80]. Both specific and cooperative actions of proteoglycans and EAAT1s of astrocytes at NRs contribute to extracellular glutamate regulation, NR maintenance, and stability of Na_v_ aggregation. These features are crucial for adequate propagation of action potentials. Thus, if glial processes, associated ECMMs and EAAT1s are altered in depression they could critically participate in pathophysiological mechanisms involving disturbances of connectivity among brain centers in MDD.

The processes of astrocytes associated with NRs, in conjunction with the myelin of oligodendrocytes, regulate the speed of saltatory conduction, thus allowing optimal communication between neurons [64,81]. Alterations of astrocyte processes abutting NRs and their vicinity could result in abnormal signal conduction and WM pathology as revealed by neuroimaging in depression [7,60,82–89]. Recently, abnormal structure and protein expression of axonal and oligodendrocyte proteins at NRs and adjacent axon segments were reported in corpus callosum and frontal cortex of chronically stressed rodents [61], suggesting that pathology at NRs may occur in MDD. So far it is unknown whether pathological alterations in astrocyte processes and NR-associated proteins in models of depression or in humans are direct participants in the etiology of depression. However, our previous research in astrocytes in MDD and our mRNA studies on WM would suggest that NR-related astrocytes changes contribute to depression-like dysfunction [17,72]. The possible involvement of astrocyte processes at NRs in WM functional and structural changes is consistent with reduced GFAP immunoreactive area fraction of processes and levels that we found in relatively young MDD subjects [53,90] Since repeated stress is a risk factor for MDD [91] structural and molecular alterations associated with astrocyte processes at NRs may occur in prefrontal brain regions of MDD patients. Such pathology associated with astrocyte processes at NRs should be taken into account to explain the pathophysiology of depression or design innovative approaches for the treatment of affective and stress-related disorders.

## ASTROCYTES AND THE ROLE OF NEUROTROPHIC FACTORS

The importance of astrocytes to the maintenance of adequate signal conduction along axons also includes their role as providers of neurotrophic factors to facilitate oligodendrogenesis and myelination after damage to the white matter [92,93]. Other researchers have shown that expression of neurotrophins by astrocytes, and their receptors in oligodendrocytes, is regulated by increased exercise [94], further suggesting that repair and plasticity of axons and their myelin is greatly influenced by neurotrophin release from astrocytes [93] even in non-pathological plastic processes. By contrast, in particular conditions involving inflammatory and toxic signals, and the mediation of neurotrophin receptors, dysfunctional astrocytes are potential enhancers of copper distribution and other deleterious compounds, and thus increase the possibility of myelin damage [93,95]. Other research has shown that myelination and the interaction between oligodendrocyte myelin membranes and axons at the paranodes lining NRs is dependent on astrocyte gene expression regulation by epigenetic factors such as Methyl-CpG-Binding Protein 2 (MeCP2), which in fact regulates the expression of growth factors such as BDNF and NGF by astrocytes [96]. The involvement of astrocyte-derived neurotrophic factors and their receptors in WM may be of great relevance to understand the contribution of neurotrophins to connectivity disturbances in depression since neurotrophic deficit or upregulation (depending on the brain area) have been found in the brains of affective disorder patients and in animal models of stress [97,98]. For example, significant correlation of neurotrophin DNA methylation and reduced WM integrity (assessed by neuroimaging methods) has been found in the WM of frontal bran regions in major depression [99]. Most importantly, the altered methylation patterns in depression subjects are strongly associated with astrocyte-related dysfunction [100]. Also in prefrontal brain regions, diffusion tensor magnetic resonance imaging (DTI) has shown increased fractional anisotropy in WM fiber bundles associated with particular genotypes of the neurotrophic tyrosine kinase receptor type 2 (*NTRK2*), but only in subjects diagnosed with depression [101], while specific polymorphisms of the BDNF gene are associated with detection of WM DTI disturbances in subjects with high depression severity [102,103].

## CONCLUDING REMARKS

Morphological and neurochemical differences of WM astrocytes as compared to most GM astrocytes, their ubiquitous involvement in myelin formation and maintenance, and their crucial association with nodes of Ranvier to support the regeneration of action potentials, suggest a critical contribution of those aspects of astrocyte biology to the integrity and fidelity of nervous signals carried along WM axons, and thus to normal brain connectivity. Accordingly, disturbances in brain connectivity that have been described in depression and other psychiatric disorders may be at least partially a consequence of disturbances in astrocyte interactions with oligodendrocyte membranes and nodes of Ranvier in the involved cortical brain regions. An argument could be made that targeting astrocyte-related pathology in the white matter may provide new or complementary approaches to better therapies that improve connectivity in the brain of patients with affective and other psychiatric disorders.

## Data Availability

The dataset of the study is available from the authors upon reasonable request.
